# Efficacy and efficiency of a new therapeutic approach based on activity-oriented proprioceptive antiedema therapy (TAPA) for edema reduction and improved occupational performance in the rehabilitation of breast cancer-related arm lymphedema in women: a controlled, randomized clinical trial

**DOI:** 10.1186/s12885-020-07558-x

**Published:** 2020-11-09

**Authors:** María Nieves Muñoz-Alcaraz, Luis Ángel Pérula-de-Torres, Jesús Serrano-Merino, Antonio José Jiménez-Vílchez, María Victoria Olmo-Carmona, María Teresa Muñoz-García, Cruz Bartolomé-Moreno, Bárbara Oliván-Blázquez, Rosa Magallón-Botaya

**Affiliations:** 1 Córdoba and Guadalquivir health district, Andalusia Health Service, Córdoba, Spain; 2 Andalusia Health Service, Maimonides Institute of Biomedical Research of Cordoba (IMIBIC)/Universidad de Cordoba, Córdoba, Spain; 3 Valle de los Pedroches Hospital, Pozoblanco, Spain; 4 Reina Sofia Hospital, Córdoba, Spain; 5 Institute for Health Research Aragon (IIS Aragon), Zaragoza, Spain; 6 Aragones Heath Service, Zaragoza, Spain; 7 University of Zaragoza, Zaragoza, Spain

**Keywords:** Occupational therapy (OT), Breast cancer (BC) - lymphedema (L), Activity-oriented proprioceptive antiedema therapy (TAPA in Spanish), Health-related quality of life (HRQOL), Complex decongestive therapy (CDT)

## Abstract

**Background:**

Breast cancer (BC) is a major public health issue. More than one out of five women treated for breast cancer will develop lymphedema in an upper extremity. Current evidence advocates transdisciplinary oncological rehabilitation. Therefore, research in this area is necessary since limited consensus having been reached with regard to the basic essential components of this rehabilitation.

Consensus has, however, been reached on the use of decongestive lymphedema therapy (DLT), but due to a lack of tests, the necessary dosages are unknown and its level is moderately strong.

This study attempts to verify both the efficacy of activity-oriented proprioceptive antiedema therapy (TAPA), as compared to conventional treatments such as DLT or Complex Physical Therapy (CPT), as well as its efficiency in terms of cost-effectiveness, for patients affected by breast cancer-related arm lymphedema.

**Methods:**

Controlled, randomized clinical trial with dual stratification, two parallel arms, longitudinal and single blind. 64 women with breast cancer-related arm lymphedema will take part in the study. The experimental group intervention will be the same for stage I and II, and will consist of neuro-dynamic exercises oriented to the activity, proprioceptive neuromuscular facilitation activities and proprioceptive anti-edema bandaging. The control group intervention, depending on the stage, will consist of preventive measures, skin care and exercise-prescribed training in the lymphedema workshop as well as compression garments (Stage I) or conservative Complex Decongestive Therapy treatment (skin care, multi-layer bandaging, manual lymphatic drainage and massage therapy) (Stage II).

**Results:**

Sociodemographic and clinical variables will be collected for the measurement of edema volume and ADL performance. Statistical analysis will be performed on intent to treat.

**Discussion:**

It has been recommended that patient training be added to DLT, as well as a re-designing of patient lifestyles and the promotion of health-related aspects. In addition, clinical trials should be undertaken to assess neural mobilization techniques and proprioceptive neuromuscular facilitation should be included in the therapy. Cohesive bandaging will also be performed as an early form of pressotherapy. The proposed study combines all of these aspects in order to increased comfort and promote the participation of individuals with lymphedema in everyday situations.

**Limitations:**

The authors have proposed the assessment of the experimental treatment for stages I and II. One possible limitation is the lack of awareness of whether or not this treatment would be effective for other stages as well as the concern for proper hand cleansing during use of bandages, given the current COVID-19 pandemic situation.

**Trial registration:**

This trial was registered in ClinicalTrials.gov (NCT03762044). Date of registration: 23 November 2018. Prospectively Registered.

## Background

Breast cancer (BC) is a major public health issue, being the most common global cause of cancer-related deaths in females and the second most common form of cancer, with over 2 million new cases occurring in 2018 [1]. BC survival rates have improved notably over the past 20 years (survival from this type of tumor increases by 1.4% annually) [2]. BC survivors face challenges in their activities of daily living (ADL), as a result of both the illness and the subsequent treatments undertaken. These challenges include altered sensory and pain functions and neuromusculoskeletal and movement-related alterations, as well as alterations of the skin and related structures. These symptoms tend to continue for extended periods of time and therefore, BC survival has been categorized as a chronic illness with new challenges arising for rehabilitation services [3, 4].

It is estimated that more than one out of every five women treated for BC will develop lymphedema in an upper extremity (20% in 6 months, 36% in 1 year and 54% within 36 months). The most relevant risk factors are the number of lymph nodes excised in surgery, radiation therapy and obesity. This is a chronic and progressive disorder that causes edema, skin changes (which may cause fibrosis and deformity), pain, sensitivity alterations, fatigue, limitations in joint rotation, recurrent infections, an inability to independently carry out ADL, low self-esteem, etc. Although it has been recognized for years now, consensual treatment dose programs have yet to be established [5].

Tertiary prevention in BC attempts to combat the consequences of this illness. It includes aspects such as rehabilitation, relapse prevention, early diagnosis of second malignancies, management of late complications through treatments and emotional support. This type of assistance has been traditionally presented for some time now in specialized health care, but it is currently becoming an increasing primary care responsibility [6, 7].

Rehabilitation of BC patients is intended to ensure their social integration and participation. Current evidence suggests the importance of transdisciplinary oncological rehabilitation. Therefore, research in this area is essential [8], since it is of great importance and quite different from the treatment phase and should include physical and occupational therapies (OT) [9]. Numerous rehabilitation models currently exist, but consensus has not been reached as to the basic necessary components of integral rehabilitation for cancer survivors [10, 11]. The regional government of Andalusia (Spain) designed an Integrated Palliative Care Process (IPC) for BC [12], which includes aspects to be carried out during the rehabilitation process, including lymphedema therapy, whose implementation has been quite disparate in the distinct provinces of the region. The literature also finds follow up recommendations based on risk factors and care level [7].

Consensus has been reached with regard to the utility of decongestive lymphedema therapy [13] but tests have yet to determine the optimal treatment dosage to be used and the level of evidence regarding its effectiveness in lymphedema treatment is only moderately strong, due to a limited number of randomized trials conducted with control groups, a lack of well-controlled interventions and measurements of precise volume, mobility or function and quality of life [5, 14, 15]. Similarly, the prescription of compression garments for lymphedema is quite varied and may be due to the lack of underlying evidence to inform on the treatment [16–18].

Evidence also suggests that complete and effective post-operative BC rehabilitation should also include patient self-control (empowerment), focus on the redesigning of their life style and include health promotion aspects [8, 19–21].

Studies have also confirmed that women with lymphedema and pain following BC may present alterations in their neuro-mechanical sensitivity [22] and neuropathic pain, as a result of nerve compression, or peripheral neuropathy induced by chemotherapy [2]. So, clinical trials should be carried out to assess neural mobilization techniques that focus on the functionality of the upper limbs [23], based on the clinical neural mobilization mechanism to reduce the intra-neural edema [24].

Studies have suggested the incorporation of proprioceptive neuromuscular facilitation elements (PNF) with traditional methods of manual lymphatic drainage (MLD), to facilitate the treatment process for lymphedema patients, since they induce powerful synergic effects on edema volume, range of motion (ROM) of the shoulder, pain and depression [25]. Their independent or combination use with laser treatment has also been suggested, since improvements were also found in these variables and even in the rate of blood circulation in the underarm [26]. Also, in a study in which bandaging was not used, CDT was combined with PNF and respiration, reducing the lymphedema and also improving ROM [27], which could potentially improve activity performance, participation level and thus, quality of life [14, 28].

In response to the evidence regarding the use of the Coban®3 M bandage as an early precursor to pressotherapy in the rehabilitating treatment of lymphedema to reduce prolonged edema (the causal agent of multiple complications and functional loss) [18, 29–31], we propose the possibility of testing the effectiveness of similar bandages, but ones that avoid compressive syndromes, decrease pain and edema, promote the development of ADL and improve the treatment’s cost-effectiveness [32, 33]. We suggest combining its use with the three previously mentioned techniques (healthcare education/patient training, neuro-dynamic activities geared to neuro-muscular use and proprioceptive neuro-muscular facilitation oriented towards ADL) and which, collectively, are referred to as “TAPA” (in Spanish, Activity-oriented proprioceptive antiedema therapy).

Given the consensus for conservative lymphedema treatment, such as Complex Decongestive Therapy (CDT) or Complex Physical Therapy (CPT), we believe that comparison with our TAPA treatment should be made with this type of treatment.

This study attempts to assess the effectiveness of TAPA, in terms of cost-effectiveness, calculating costs of material and human resources. Current expenditure on bandaging materials for CDT is at least double that which would be spent on TAPA, since, instead of multi-layers (usually two or three) only one is used here, and patients may require an annual prescription for compression garments, included in the catalogue of orthoprosthetic products of the Andalusian Health Service [34] and the use of these is minimized by TAPA. Personnel expenses for CDT are for approximately 60 min per treatment, using an individual approach formula, while TAPA sessions are approximately 30 min, and are carried out individually, with the possibility of a group approach. This, as well as the effectiveness of the treatment in terms of measurement of the reduced volume of the lymphedema, will improve the functionality of the upper extremity, during the performance of daily living activities (occupational) and therefore, in HRQOL.

## Hypotheses and objectives

Hypothesis: Activity-oriented proprioceptive antiedema therapy is more cost-effective than standard treatment.

### Objectives

This study aims to verify both the efficacy of the new therapy (TAPA) versus conventional treatment, as well as its efficiency in terms of cost-effectiveness, in patients suffering from Breast Cancer-Related Arm Lymphedema (BCRAL).

### Specific objectives

- To measure the decrease in volume of the affected limb treated with TAPA and the maintenance of the effect over time (1 month and 3 months).
- To evaluate its effect on the performance of activities of daily living (ADL).
- To evaluate its effect on health-related quality of life (HRQOL).
- To determine efficiency in terms of simplicity of handling and economy of the anti-edema bandage technique used in the TAPA group.

## Methods

### Design

Controlled clinical trial, randomized by stratification in two gradients, with two parallel arms, longitudinal and single blind. To measure the efficiency of the new treatment, a cost-effectiveness study will be performed [34–36] .

### Study subjects

The study population will be females who were treated for breast cancer, diagnosed with Breast Cancer-Related Arm Lymphedema (BCRAL), referred to the UGC Rehabilitation Inter-levels of the Reina Sofía Hospital–Córdoba and Guadalquivir health district, from either the multi-disciplinary breast department of Specialized Care or the Primary Care teams, whose flow diagram of referrals to rehabilitation, both in-hospital and at home, for the treatment of post-mastectomy lymphedema and for individual or group treatment, is described in the Guide to Rehabilitation and Physical Therapy Procedures in Primary Care [37].

### Selection criteria

#### Inclusion criteria

- Women, treated for BC with a diagnosis of level I and II BCRAL, according to stratification of the Work Group of the 11th International Congress of Lymphology [38], which establishes:

• Stage 1 or mild: difference in the circometry of less than 4 cm (in volume 10–25%) with respect to the healthy arm.

• Stage 2 or moderate: difference in the circometry of between 4 and 6 cm (in volume 25–50%) with respect to the healthy arm.

- Patient’s informed consent.

#### Exclusion criteria

- Health problems, illnesses or disabilities that prevent them from participating in the intervention.
- Other pathologies affecting the HRQOL: untreated cardiac, renal or respiratory insufficiency.
- Bi-lateral lymphedema.

#### Participant inclusion

Department specialist in rehabilitation invites the patient to participate, informing them of the purposes of the research and its procedures.

If they decide to participate, they should sign the Informed Consent, which shall be kept by the researcher.

Once the subject is included in the study, they will be assigned a study protocol number, which will serve to identify them in all of the study documents. This is a unique identification number created with an algorithm based on the center where they came from, their initials and date of birth, which shall subsequently be encrypted in an 8-digit number in the coordinating center. The codes shall be delivered to each researcher at the start of the study.

### Sample size

It is agreed that a decrease in the volume of the clinically relevant lymphedema may be established at 150 ml. (minimal detectable value) [39], 20% with respect to the baseline. Based on data from the literature on means and standard deviations [25, 40], for an alpha error of 0.05 and a statistical power of 0.80, the necessary sample size would be 29 subjects per group (calculation performed with EPIDAT 4.2). It is assumed that the standard treatment effect in the control group will lead to a reduction in arm volume by an average of 5%, the treatment effect in the experimental group will cause an average reduction in arm volume of 20%, and the standard deviation will be similar in both groups, approaching 20% [41]. Taking into account the dropout rate of 10% in each group, the corrected sample size is 64 patients, randomly assigned to two groups with 32 patients each.

### Randomization

Subjects were selected randomly using stratified random selection, in stages I or II, according to stratification of the Work Group of the 11th International Congress of Lymphology. Following capture and recruitment, participants were randomly assigned to one of the two groups **(experimental or control).**

### Random allocation and concealment of allocation

The random assignment sequence was created using EPIDAT, 3.1 statistical software, which stratifies the patients according to lymphedema stage, with a 1:1 ratio, using blocks having a random size of 4. The assignment sequence was hidden from the researcher assessing the participants through sequentially numbered envelopes that are opaque, sealed and stapled.

Allocation will be carried out by a department specialist in the Physical Medicine and Rehabilitation department.

### Sampling technique

Consecutive sampling. Patients who comply with the selection criteria will be invited to participate in the study as they are identified and captured by the field researcher entrusted with this task.

Sampling will be carried out by a department specialist from the Physical Medicine and Rehabilitation department.

### Blinding

Following the assignment of the interventions, these results will be hidden from the evaluator, who is a final year medical resident specializing in Physical Medicine and Rehabilitation (AJ), who will also monitor the data and data analysts (LA and J), who will not receive information on which of the treatments corresponds to the distinct data.

The party responsible for the random assignment and participant capture will inform the evaluator and the participants of the distinct dates of results assessment for each of them, and they will be reminded of the same and will have this information confirmed (or modified) via telephone on the preceding days.

Participants will attach the protocol follow-up sheet, registering any adverse effects, if occurring, as well as drop outs, losses and causes for the same.

Study revision by part of the research team is ongoing, through the use of the new technologies and a formal quarterly meeting.

### Study variables and measurement instruments

The study variables are shown in Tables 1, 2, 3.

**Table 1:** Independent study variables and their measurement instruments

INDEPENDENT VARIABLES (Sociodemographic and clinical variables)	MEASUREMENT INSTRUMENTS (to be filled in with information from interviews and clinical records)
- ID: Identification number for the study (1, 2, 3...)	ID:
- Age.	Date of bird _/_/_ _ years old
- Group (Treatment carried out):	TAPA / standard.
- Work activity:	Working (Which?) /Retired/Unemployed/Others (Which?)
- Menstrual situation:	Pre-menopausal/Post-menopausal
- Laterality:	Right/ left/Both
- Body mass index (BMI): - Obesity:	BMI: height/weight^2^ yes (BMI > =30)/ no (BMI < 30)
- AHT (Arterial hypertension):	Yes (> = 120/80 mmHg/take medication) / no (< 120/80 mmHg)
- DLP (Dyslipidemia):	Yes (> = 200 mg dl/take medication) / no (< 200 mg/dl)
- DM (Diabetes):	Yes (> = 126 mg dl/take medication) / no (< 126 mg/dl)
- Heart disease:	Yes / no
- Kidney failure: Yes / no	Yes / no
- Vascular disease: Yes /no	Yes / no
- Thyroid disease: Yes / no	Yes / no
- Pacemaker: Yes / no	Yes / no
- Electro-stimulator: Yes /no	Yes / no
- Date of BC diagnosis	_/_/_
- Date of lymphedema diagnosis	_/_/_
- Stage:	I, II
- Edema location:	Hand(H)/Forearm (F)/Arm (A)/ Breast H + F/ H + F + A/ F + A/ Complete/ Others (this category should be thoroughly considered)
- Breast:	Right/ left
- Date of surgical intervention:	_/_/_
- Type of tumor: - Intervention type: Conservative/mastectomy	ductal infiltrating/in situ/ lobular/ inflammatory/tubular/other (which?): Conservative/mastectomy
- Axillary surgery:	sentinel ganglion biopsy (SGB)/ lymphadenectomy /Both/none
- No. of excised ganglia:	N°
- No. positive ganglia:	N°
- Complications:	Infection/ hematoma/ seroma/nerve lesion/Axillary web syndrome
- Chemotherapy:	Yes / no
- Radiotherapy:	Yes / no
- Hormone therapy:	Yes / no
- Immunotherapy:	Yes / no
- Breast reconstruction:	Yes / no Yes: - Type of reconstruction: Prosthesis/back/ abdomen - Reconstruction time: Immediate/ deferred
- Date of symptom onset:	_/_/_
- Lymphedema:	Right/ left

**Table 2:** Primary dependent study variables and their measurement instruments

PRIMARY DEPENDENT VARIABLES	MEASUREMENT INSTRUMENTS/INTERPRETATION OF RESULTS
- Treatment adherence:	- yes/no Calculation of the percentage of treatment compliance for therapeutic adherence. PC = number of activities performed by patient/number of activities prescribed by the therapist *100. If PC is equal to or over 80%, it is considered that there was good treatment adherence. If less, no.
	-Physical state of the limb (inspection and palpation of the affected area). Photographs will be taken of the limb [42].
- Skin hydration: - Skin lesions: - Nail lesions: Yes / no - Induration / Fibrosis: Yes / no - Pitting edema: Yes / no - Stemmer: Yes / no	Normal/ dry/sweating Yes / no Yes / no Yes / no Yes / no Yes / no
	-Visual analogue scale (VAS), where 0 is no pain, heaviness or tension and 10 is maximum pain, heaviness or tension.
- Pain: - Heaviness: - Tension:	VAS 0–10 VAS 0–10 VAS 0–10
- Shoulder joint rotation:	Degrees with goniometer: Flexion___/Abduction___/External rotation___
	-Volume, through circometry (manual measurement of perimeters of the limbs with a tape measure, the value of the volume is approximate and volume is calculated using the Kuhnke formula Vol = (C1 2 + C2 2 + … Cn 2)/ π)) [43–45].
- Degree of lymphedema (according to % of volumetric):	_Grade 1 or mild: difference in circometry less than 4 cm (in volume 10–25%) with respect to the healthy arm. _Grade 2 or moderate: difference in circometry between 4 and 6 cm (in volume 25–50%) with respect to the healthy arm.
- Volumetric %: - Difference in volume in % between both extremities: - Volumetric in ml:	Vol = __% D = __% Vol = __ml *an edema reduction of 150 ml over the basal value is required in order to be considered clinically relevant
- Function of the upper limb/activity performance.	- Value of Quick Disabilities of the Arm, Shoulder and Hand (Quick DASH) [21], with transcultural, reliability, validity and sensitivity adaptation to changes of its extended version in 2006 [46, 47]. - Quick DASH value = __% (the result is calculated as a percentage, the higher the obtained result, the greater the disability or symptom)
- Treatment costs	Sum of expenses: -Prescription of compression garment: Yes (specify type and cost ______________) / NO - Type of bandaging used: proprioceptive / multi-layer (number of rolls used per price of each of the same) - Total number of treatment hours with therapist: 5/10/other (which:___) (number of hours used per professional hour price)

**Table 3:** Study secondary dependent variable and its measurement instruments

SECONDARY DEPENDENT VARIABLE	MEASUREMENT INSTRUMENTS/INTERPRETATION OF RESULTS
-Health related quality of life (HRQOL).	- Value of Upper Limb Lymphedema 27 (ULL-27), specific instrument for patients with Breast Cancer-Related Arm Lymphedema, transculturally adapted and validated in its Spanish version in 2016 [48]. (The result is calculated as a percentage of social, physical and psychological dimensions in which the higher the result obtained, the greater the disability or symptom/poorer the HRQOL). - Health Questionnaire EQ-5D-5L by EuroQol Group, generic and standardized instrument used to describe and value the HRQOL of a group or population and validated in Spain by Badia X. et al. in 1999 [49]. Numeric value between 3 and 45, in which the higher the score, the poorer the HRQOL.

### Information sources

A data collection notebook/form will be created, which will be accompanied by a procedures manual.

The following instruments will be used for the measurement (Table 2), comparison and treatment of dependent variables:

### Statistical analysis

Analysis is performed for intent to treat. Data lacking after the basal measurements are replaced using the last available observation. Quantitative and qualitative variables will be described as usual (mean, standard deviation; proportions). An analysis of basal comparability of the sociodemographic and clinical variables **(**Table 1**)** will be carried out between both treatment groups. Then, a bivariate analysis will be conducted, previously verifying that the quantitative variables follow a normal distribution, using the Kolmogorov-Smirnov or Shapiro-Wilk tests, in which case, a repeated measures ANOVA is carried out (Mixed Models), to analyze the profile of patients from the basal measurement (24–72 h before treatment onset), after completing 10 sessions and one and 3 months later. If the variables do not follow a normal distribution, non-parametric tests will be used (Friedman’s test). In addition, a multivariate analysis will be performed, as well as a survival analysis, Cox regression or multiple linear regression, taking into account the examined sociodemographic and clinical covariables resulting statistically significant following the bivariate analysis, for their inclusion in the model or to be controlled for, given their potential confounding effect. The main dependent variable will be the reduction of volume of the affected extremity. Statistical analysis will be performed using the Statistical Package for the Social Sciences (SPSS v.17). Statistical significance values will be those normally used (*p* < 0.05) and bilateral contrasts will be carried out. To evaluate effectiveness, a comparative analysis of economic costs of both treatments will be performed.

### Interventions

Following capture and recruitment, participants were randomly assigned to one of the two groups.

Both groups will receive a 3-h long healthcare education workshop on lymphedema (Lymphedema workshop), led by a department specialist in the Physical Medicine and Rehabilitation Dept. of the Clinical Inter-level Rehabilitation Management Area (MV), and offering basic knowledge on the physiopathology of lymphedema, identification of symptoms, preventive skin care measures, ADL orientation, including physical activity and recommendations for neck and arm anti-edema exercises and postures to be carried out at least once daily.

The interventions used for the Experimental group will be the same for stage I and II, and will receive 10 sessions (2 per week), of 30 min each, led by an Occupational Therapist from the Córdoba and Guadalquivir health district (MN):

− 10-min neuro dynamics oriented to the activity.

− 10-min proprioceptive neuromuscular facilitation activities.

− 10-min proprioceptive anti-edema bandaging, including patient and/or caretaker instructions for placement, as well as assistance on techniques to use for performing everyday activities.

Proprioceptive anti-edema bandaging will be placed after carrying out the neuro dynamic and neuromuscular facilitation activities. Cohesive elastic bandages will be used, containing a high content of cotton and anti-slip.

The Control group intervention, depending on the stage, will be led by a physiotherapist from the Reina Sofía Hospital in Córdoba, and will consist of:

Stage I:

- Preventive measures, skin care and exercise prescribed by the rehabilitation physician and training in the lymphedema workshop.

- Compression garments.

-Duration of 5 weeks.

Stage II:

Ten sessions of 60 min of conservative treatment of Complex Decongestive Therapy (the literature does not refer to specific protocols to quantify session duration, therefore, expert opinions based on everyday practice were used as a reference):

-Skin care.

-Multi-layer bandaging.

-Manual lymphatic drainage.

-Massage therapy (22).

Participants will be notified that during the intervention and follow-up period, they may not undertake other treatment for their BCRAL and, in the case of being subject to experimental treatment and not achieving the expected edema reduction, they may complete standard treatment upon study completion.

**Fig. 1:**
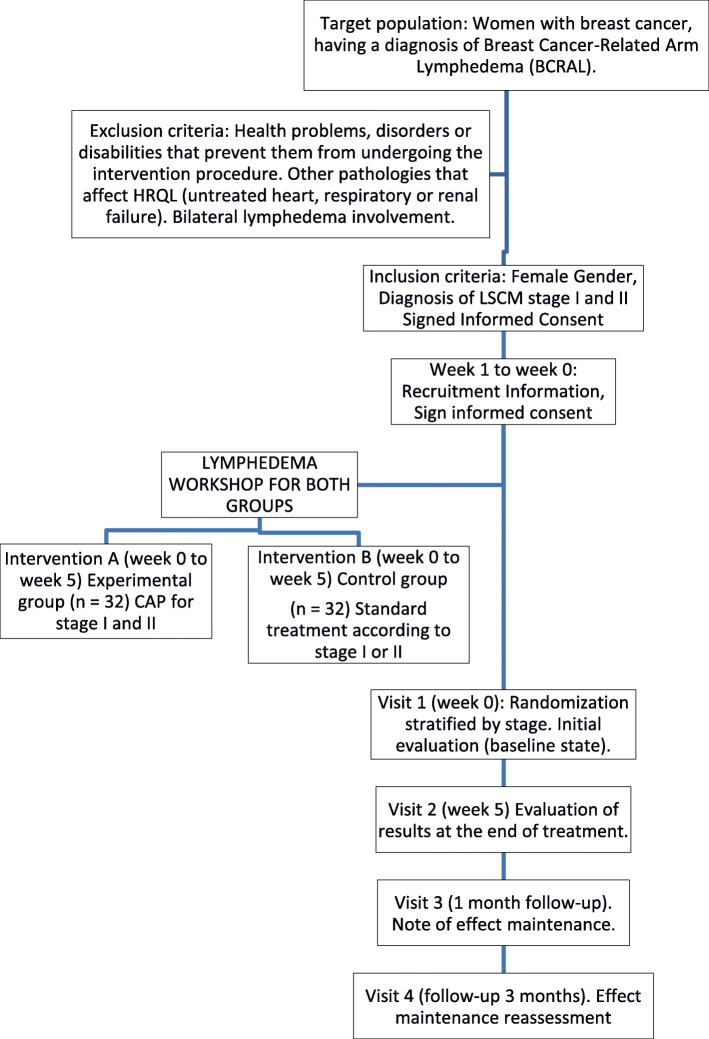
Study flow design

To analyze the patient profile from the basal measurement (24–72 h prior to treatment onset), until immediately following completion of the 10 sessions and one and 3 months later (Fig. 1).

## Study duration

**Fig. 2:**
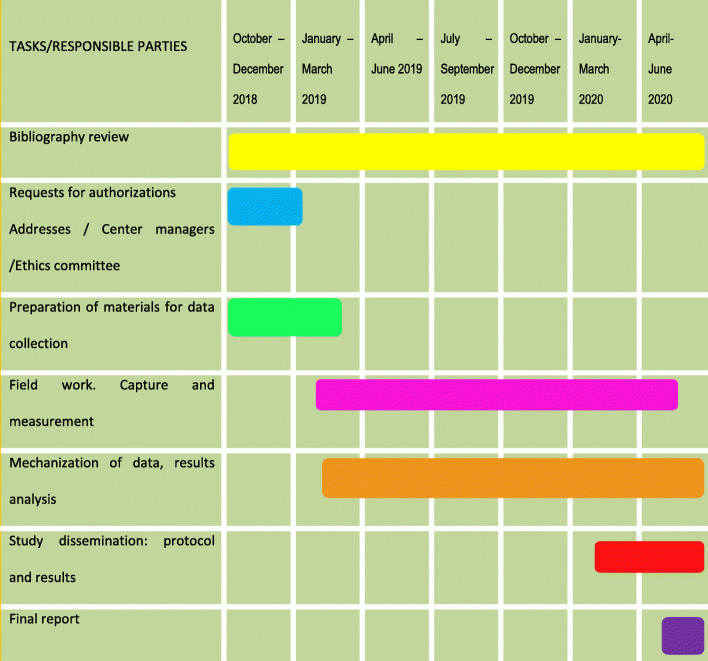
Gantt’s study diagram

The anticipated study duration is 18 months, which may be modified based on the patient recruitment period. In Fig. 2, Gantt’s study diagram is shown.

## Expected results

For the desirable data following data analysis, the experimental treatment would include the following:

- Reduce the volume of the affected extremity by at least 150 ml. more than the control group intervention.
- Positively impact the treated patient’s HRQOL, improving the mean scores of the three dimensions from the ULL-27 questionnaire by at least 12.58 points (minimum detectable change).
- Improve the functionality of the upper limbs affected by lymphedema, measured using Quick Dash on at least 15 points.
- Reduce costs by at least 50% with respect to standard treatments in the respective stage.

## Discussion

The experimental TAPA treatment attempts to incorporate the advantages of comfort with respect to standard treatment by substituting compressive elements for proprioceptive ones, which facilitate the participation of the individuals suffering from the lymphedema in everyday situations.

It is conceptualized as a patient-focused, personalized treatment, in which the significant prescribed activities adapt to each case.

Based on the study’s anticipated results, TAPA may result in the creation of the first lymphedema rehabilitation protocol in an upper limb, secondary to breast cancer, which reduces intervention costs.

The recommended occupational therapy contribution based on evidence from lymphedema rehabilitation, suggests the inclusion of the occupational needs of the attended individuals and the adaptation to their everyday surroundings, potentially improving their self-perception of the health-related quality of life.

## Data Availability

Not applicable.

## Abbreviations

ADL: Activities of daily living
TAPA: Activity-oriented proprioceptive antiedema therapy (In Spanish: Terapia antiedema propioceptiva orientada a la actividad)
BC: Breast cancer
BCRAL: Breast cancer-related arm lymphedema
CPT: Complex physical therapy
CDT: Complex decongestive therapy
DLT: Decongestive lymphedema therapy
HRQOL: Health-related quality of life
MLD: Manual lymphatic drainage
OT: Occupational therapy
PC: Primary care
PNF: Proprioceptive neuromuscular facilitation element
ROM: Range of motion
TNM: Techniques of neural mobilization
